# Gender differences in prescribing patterns for patients with Parkinson’s disease in Japan: a retrospective observational study using insurance claims databases

**DOI:** 10.3389/fneur.2025.1571718

**Published:** 2025-07-04

**Authors:** Morinobu Seki, Yayoi Kawata, Ayako Hayashi, Shinji Fujimoto

**Affiliations:** ^1^Department of Neurology, Keio University School of Medicine, Tokyo, Japan; ^2^Parkinson's Disease Center, Keio University Hospital, Tokyo, Japan; ^3^Japan Medical Office, Takeda Pharmaceutical Company Limited, Tokyo, Japan

**Keywords:** aged, Japan, longitudinal studies, Parkinson’s disease, practice patterns, physicians’, sex factors

## Abstract

**Background:**

Parkinson’s disease (PD) is a neurodegenerative disorder, with increasing prevalence among aging populations. Gender differences in PD extend to symptom presentation and treatment response, suggesting the need for gender-specific management strategies.

**Methods:**

This gender-stratified analysis of a retrospective observational study used data from three nationwide Japanese healthcare databases. Patients aged ≥30 years diagnosed with PD between June 2016 and May 2021 were included. Patient demographics, prescribing patterns, and levodopa dosages were analyzed descriptively.

**Results:**

Of 39,731 patients with PD identified, females (*n* = 22,724) outnumbered males (*n* = 17,007), especially in the ≥75 years group. Levodopa was the most commonly prescribed drug for both genders. The mean ± standard deviation maximum levodopa dose was numerically higher in males (520.0 ± 426.8 mg) compared with females (498.7 ± 424.2 mg). Usage of monoamine oxidase B inhibitors (MAOBI) was 24.0% in males and 18.9% in females. Among newly treated patients, >70% of both genders started treatment with levodopa monotherapy; a slightly higher proportion of males tended toward levodopa combination therapy. For both genders, concomitant drugs were most commonly MAOBI, non-ergot dopamine agonist, and zonisamide. However, females tended to receive a more diverse range of medications than these three drugs.

**Conclusion:**

This analysis highlights the high number of elderly female patients with PD in Japan. Slight gender differences in maximum levodopa dose and prescriptions for anti-PD drugs were observed. These findings emphasize the importance of personalized treatment approaches in PD management considering gender-specific differences in drug efficacy and side effects.

**Clinical trials registration:**

https://center6.umin.ac.jp/cgi-open-bin/ctr_e/ctr_view.cgi?recptno=R000053425, identifier UMIN000046823.

## 1 Introduction

Parkinson’s disease (PD) is a neurogenerative disorder characterized by the gradual onset of motor dysfunction, with its prevalence increasing with age, particularly around 65 years and older (1–4). In Japan, the prevalence of PD is estimated to be approximately 100–180 individuals per 100,000 population (5), and this number is expected to rise rapidly as the population continues to age (2). While PD prevalence is generally comparable between males and females globally, females in Japan have been reported to exhibit a higher prevalence (5).

Gender differences in PD extend beyond prevalence and include variations in symptom presentation and treatment responses (6). Males with PD are more likely to experience rigidity and gait disturbances, whereas females tend to present with dyskinesia and tremor (6, 7). Additionally, females more commonly report pain, depression, and anxiety, while males are more prone to sleep disturbances and rapid eye movement sleep behavior disorder (8–11). Treatment response also varies by gender, with females showing higher bioavailability of levodopa and a greater susceptibility to levodopa-induced dyskinesias (12, 13). These differences suggest that both drug efficacy and side effect profiles may vary between genders, highlighting the need for personalized treatment strategies in PD management.

Current treatment options for PD primarily include levodopa, dopamine agonists (DA), and monoamine oxidase B inhibitors (MAOBI) (14). The current Japanese guidelines recommend initiating anti-PD drugs in patients with functional disabilities without delaying treatment post-diagnosis (5, 15). For older patients (≥70–75 years), levodopa is the preferred treatment to manage motor symptoms, while DA are recommended for younger, cognitively intact patients to minimize levodopa-induced motor complications (15). As PD progresses, combined therapies are typically required (15). The guidelines do not provide specific recommendations for treatment adjustments based on gender differences.

In a previous study, we analyzed the prescription patterns of anti-PD drugs using Japanese nationwide healthcare claim databases, with a focus on patients aged ≥75 years (UMIN000046823) (16). Building on this, the current gender-stratified study aims to investigate the epidemiological characteristics and gender differences in the real-world prescribing patterns of anti-PD drugs.

## 2 Materials and methods

### 2.1 Study design

This was a gender-stratified analysis of the previously reported retrospective observational study (16). Healthcare claims data were extracted from three nationwide databases in Japan: (a) the elderly health insurance database (DeSC Healthcare, Inc., Tokyo, Japan), covering individuals aged ≥75 years, with 2.6 million registered people; (b) the Japan Medical Data Center database (JMDC Inc., Tokyo, Japan), encompassing company employees and their dependents aged <75 years, with 13.2 million registered individuals; and (c) the National Health Insurance database (DeSC Healthcare, Inc., Tokyo, Japan), which includes individuals aged <75 years who are not eligible for the elderly or Japan Medical Data Center databases, with 2.3 million registered people. Japan’s national healthcare insurance system requires all citizens to be enrolled in one of the insurance schemes, primarily consisting of the advanced elderly medical system, employee insurance (health insurance associations and mutual aid associations), and national health insurance, depending on age and employment status (17). Insurance premiums are calculated based on income, and these systems are designed to ensure that citizens can receive necessary medical services regardless of their economic situation. As of March 2021, the number of enrollees in the elderly medical system, employee health insurance, and national health insurance was 18.1 million, 28.7 million, and 28.9 million, respectively (18). The databases used in this study appropriately represent the distribution of Japan’s population, providing sufficient data to examine prescribing patterns by age and gender. The observation period spanned from June 2016 to May 2021, with the index date defined as the first diagnosis of PD within this time frame. The anti-PD drugs approved in Japan were evaluated using the corresponding Anatomical Therapeutic Chemical codes (Supplementary Table 1).

The study protocol was approved by the Research Institute of Healthcare Data Science Institutional Review Board (No. RI2021024) in Japan. Data collection and analysis were conducted in accordance with the Japanese Ethical Guidelines for Medical and Health Research Involving Human Subjects. Due to the retrospective nature of the study and the anonymization of the data, informed consent was not required. The study was registered with the UMIN Clinical Trials Registry (https://center6.umin.ac.jp/cgi-open-bin/ctr_e/ctr_view.cgi?recptno=R000053425, UMIN000046823).

### 2.2 Study population

The study population consisted of patients diagnosed with PD for at least 6 months, identified using the International Classification of Diseases 10th Revision (code G20). Eligible patients had to have received at least one type of anti-PD drug for a minimum of 6 months following the index date, with at least two anti-PD prescriptions recorded from the index date onward. Patients younger than 30 years at the index date were excluded from the analysis.

Two groups of patients were examined. The “all patients” group included those who met the inclusion criteria without meeting any exclusion criteria. The “newly initiated PD treatment patients” group consisted of those with at least 6 months of data prior to the index date, no prescriptions for anti-PD medications during this period, and no diagnosis of other neurodegenerative diseases presenting with parkinsonism or secondary parkinsonism.

### 2.3 Outcome measures

Outcome measures included patient demographics, prescribing patterns, and database characteristics. Prescribing patterns were determined using diagnosis codes, while levodopa dosages were calculated based on the daily prescribed dose documented in prescription receipts. The mean maximum levodopa dose during the observation period was calculated.

### 2.4 Statistical analysis

Descriptive statistics were used to summarize patient demographics, prescription patterns, and levodopa dosages. Continuous variables were presented as mean and standard deviation, while categorical variables were summarized as frequencies and percentages. Gender differences in prescribing patterns were also analyzed descriptively.

All statistical analyses were conducted using SAS® 9.4 (SAS Institute Inc., Cary, NC, United States) and R, Version 4.1.1 (R Foundation for Statistical Computing, Vienna, Austria).

## 3 Results

### 3.1 Study population

There were 39,731 patients with PD identified out of approximately 18 million insured individuals, including 17,007 male patients and 22,724 female patients (Supplementary Figure 1). The elderly health insurance database (*N* = 2,617,767) included 29,130 patients aged ≥75 years diagnosed with PD, with a higher representation of females (*n* = 17,578) compared with males (*n* = 11,552). Among these, 1,791 were classified as newly initiated on PD treatment, comprising 747 males and 1,044 females.

From the National Health Insurance (*N* = 2,313,264) and Japan Medical Data Center (*N* = 13,221,639) databases, a combined total of 10,601 patients aged <75 years were diagnosed with PD, with a slightly higher number of males (*n* = 5,455) compared with females (*n* = 5,146). Among these patients, 1,677 were classified as newly initiated on PD treatment, including 903 males and 774 females.

### 3.2 Age and gender characteristics

Among all patients, the number in each age group <65 years was higher in males, while more females were represented in the age group of ≥75 years (Table 1). Additionally, the mean ages showed slight variations between genders within each age group. Among patients aged ≥75 years, 79.3% of males were aged 75–84 years and 20.7% were aged ≥85 years, while 71.4% of females were aged 75–84 years and 28.6% were aged ≥85 years, showing that there were more elderly patients among the females (Table 1). Among patients who newly initiated PD treatment, the age distribution was similar to that of all patients, with an increasing number of female patients compared with male patients as age increased, resulting in a higher proportion of female patients aged ≥85 years (Table 1).

**Table 1:** Age distribution of all patients and patients who newly initiated PD treatment.

All patients	≥75 years		<75 years	
	Male (*n* = 11,552)	Female (*n* = 17,578)	Male (*n* = 5,455)	Female (*n* = 5,146)
Age, years, mean ± SD	80.6 ± 4.5	81.7 ± 5.2	60.6 ± 9.5	61.3 ± 9.5
Age group, years, *n* (%)				
≤54	–	–	1,270 (23.3)	1,052 (20.4)
55–64	–	–	1,807 (33.1)	1,594 (31.0)
65–74	–	–	2,378 (43.6)	2,500 (48.6)
75–84	9,158 (79.3)	12,555 (71.4)	–	–
≥85	2,394 (20.7)	5,023 (28.6)	–	–

Patients who newly initiated PD treatment	≥75 years		<75 years	
	Male (*n* = 747)	Female (*n* = 1,044)	Male (*n* = 903)	Female (*n* = 774)
Age, years, mean ± SD	81.0 ± 4.2	81.7 ± 4.6	61.7 ± 8.8	62.5 ± 9.2
Age group, years, *n* (%)				
≤54	–	–	173 (19.2)	135 (17.4)
55–64	–	–	324 (35.9)	225 (29.1)
65–74	–	–	406 (45.0)	414 (53.5)
75–84	582 (77.9)	765 (73.3)	–	–
≥85	165 (22.1)	279 (26.7)	–	–

PD, Parkinson’s disease; SD, standard deviation.

### 3.3 Anti-PD drug prescribing patterns by gender and age: all patients

**Figure 1:**
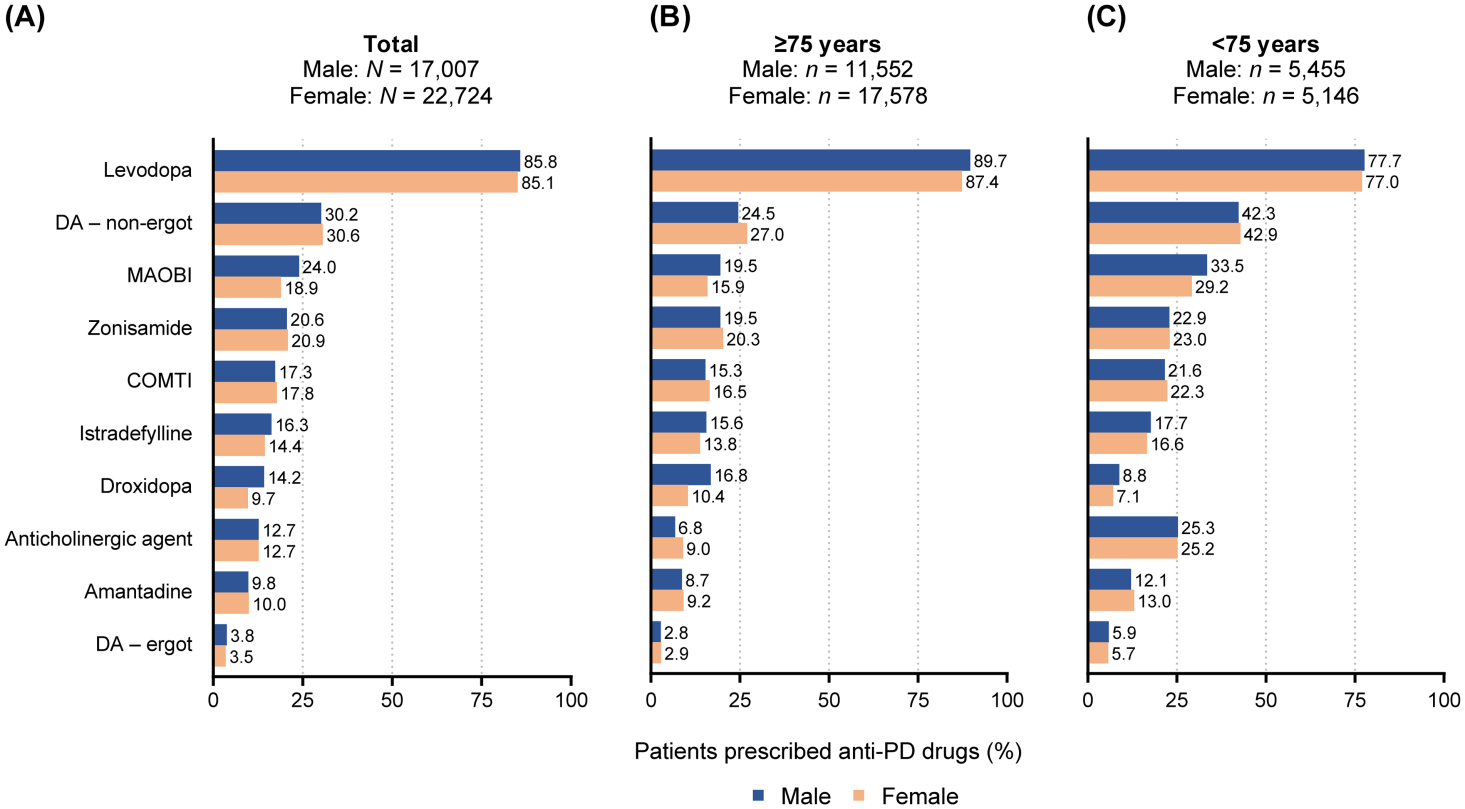
Prescribing patterns for anti-PD drugs in patients with PD by gender and age. **(A)** All patients. **(B)** Patients aged ≥75 years. **(C)** Patients aged <75 years. COMTI, catechol-*O*-methyltransferase inhibitor; DA, dopamine agonist; MAOBI, monoamine oxidase type B inhibitor; PD, Parkinson’s disease.

Levodopa was the most frequently prescribed anti-PD medication during the observation period for all patients, with a similar proportion of patients receiving levodopa prescriptions between males and females (Figure 1A). Following levodopa, males were prescribed non-ergot DA, MAOBI, and zonisamide, whereas females were prescribed non-ergot DA, followed by similar proportions of zonisamide and MAOBI. A gender difference was observed in the prescription of MAOBI and droxidopa, with a higher proportion of males receiving these prescriptions compared with females (24.0% in males vs. 18.9% in females, and 14.2% in males vs. 9.7% in females, respectively) (Figure 1A).

For patients aged ≥75 years, levodopa remained the primary drug, with a higher usage rate compared with those <75 years (Figures 1B,C). The order of the next most commonly used drugs in the ≥75 years age group was less distinct: non-ergot DA followed by similar proportions of MAOBI and zonisamide in males, and non-ergot DA followed by zonisamide and catechol-*O*-methyltransferase inhibitor in females (Figure 1B). MAOBI and droxidopa were particularly more commonly used by males (19.5% in males vs. 15.9% in females, and 16.8% in males vs. 10.4% in females, respectively), while non-ergot DA and anticholinergic agents were used slightly more by females (24.5% in males vs. 27.0% in females, and 6.8% in males vs. 9.0% in females, respectively) (Figure 1B). In patients aged <75 years, following levodopa, both males and females tended to use non-ergot DA, MAOBI, and anticholinergic agents (Figure 1C). MAOBI was used slightly more frequently in males, but other drugs were used at similar proportions between genders (Figure 1C).

**Figure 2:**
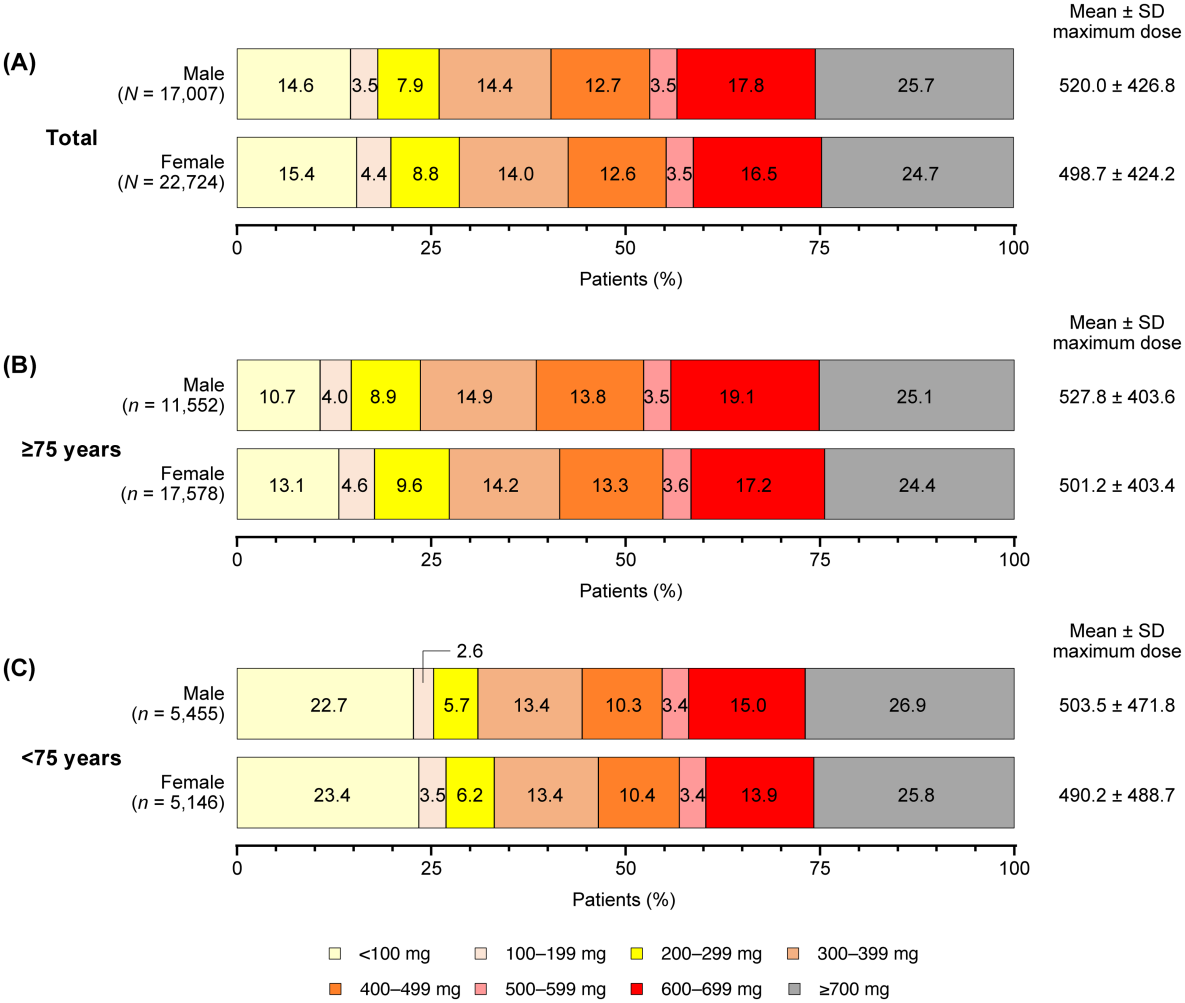
Maximum levodopa doses prescribed by gender and age during the observation period (all patients). **(A)** All patients. **(B)** Patients aged ≥75 years. **(C)** Patients aged <75 years. SD, standard deviation.

The mean maximum dose of levodopa prescribed for all patients during the observation period was numerically higher in males compared with females, with a mean ± standard deviation dose of 520.0 ± 426.8 mg in males and 498.7 ± 424.2 mg in females (Figure 2A). Subsequently, a slightly higher proportion of females in both the ≥75 years (Figure 2B) and <75 years (Figure 2C) age groups were prescribed maximum levodopa doses of less than 300 mg compared with their male counterparts. When comparing across age groups, the mean maximum dose of levodopa was slightly higher in the ≥75 years age group compared with the <75 years group for both genders. Slight variations were observed between males and females for both age groups (Figures 2B,C).

### 3.4 Anti-PD drug prescribing patterns by gender and age: patients who newly initiated PD treatment

**Figure 3:**
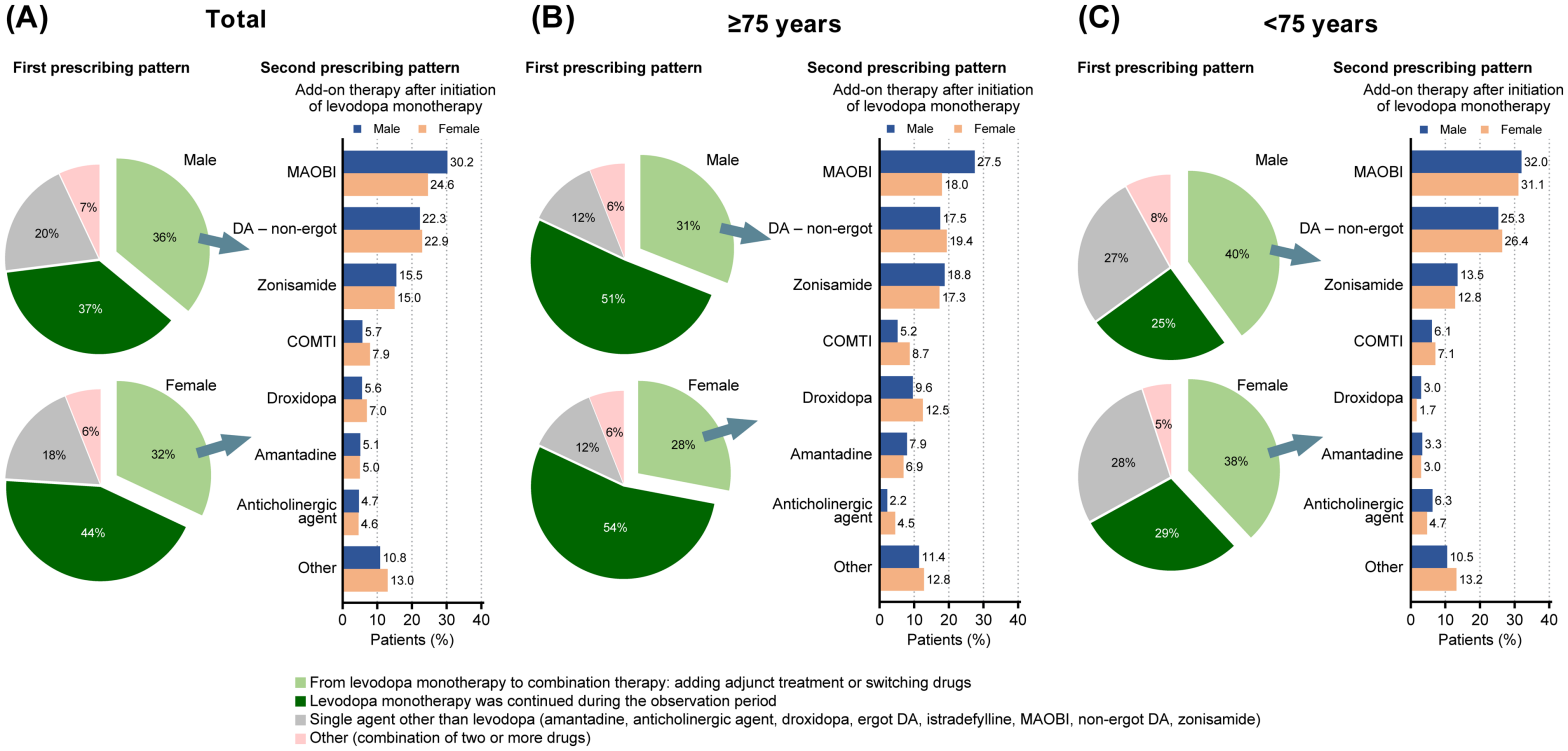
Prescribing patterns for anti-PD drugs in patients who newly initiated PD treatment, by gender and age. **(A)** All patients. **(B)** Patients aged ≥75 years. **(C)** Patients aged <75 years. COMTI, catechol-*O*-methyltransferase inhibitor; DA, dopamine agonist; MAOBI, monoamine oxidase type B inhibitor; PD, Parkinson’s disease.

In patients who newly initiated PD treatment during the observation period, over 70% of both males and females in the total population started with levodopa monotherapy, and the proportion of patients who switched to levodopa add-on therapy was slightly higher in males compared with females (36% in males vs. 32% in females) (Figure 3A). The drugs added to levodopa were most commonly MAOBI, non-ergot DA, and zonisamide for both genders, with a difference observed in the prescription of MAOBI (30.2% in males vs. 24.6% in females) (Figure 3A). Among patients aged ≥75 years, gender differences in concomitant medications following levodopa monotherapy were more pronounced. MAOBI was the most commonly prescribed drug for males (27.5%), followed by zonisamide (18.8%) and non-ergot DA (17.5%) (Figure 3B). In contrast, females in this age group were more likely to use non-ergot DA (19.4%), MAOBI (18.0%), and zonisamide (17.3%) equally (Figure 3B). While MAOBI was notably more frequently used in males compared with females, female patients had a slightly higher usage percentage of non-ergot DA (17.5% in males vs. 19.4% in females), catechol-*O*-methyltransferase inhibitors (5.2% in males vs. 8.7% in females), and droxidopa (9.6% in males vs.12.5% in females) (Figure 3B).

In contrast, for patients <75 years of age, the gender differences in concomitant medications following levodopa monotherapy were less marked (Figure 3C). Both males and females were prescribed MAOBI (32.0% in males vs. 31.1% in females), followed by non-ergot DA (25.3% in males vs. 26.4% in females) and zonisamide (13.5% in males vs. 12.8% in females) (Figure 3C).

## 4 Discussion

This gender-stratified analysis of the previously reported retrospective observational study (16) represents a novel contribution to the existing literature, being the first study to examine gender differences in PD treatment on a nationwide scale. Our findings revealed a balanced male-to-female ratio in patients with PD under 75 years; however, the ratio increased to 1.5 times higher among female patients aged ≥75 years. This aligns with prior surveys conducted by Japan’s Ministry of Health, Labour and Welfare (19). Additionally, a previous study analyzing a medical claims database from medical institutions demonstrated that significant differences in the male-to-female ratio were observed between age subgroups (*p* < 0.0001), with a higher proportion of males in the younger subgroups and a higher proportion of females in the older subgroups (aged ≥60 years) (20). This disparity may be attributed to the longevity of females and the higher prevalence of PD in the older population.

This study highlights the important gender differences in real-world prescribing patterns and dosages among PD patients in Japan. Levodopa was the most frequently prescribed treatment for both genders among all PD patients, with similar proportions of male and female recipients. However, the mean maximum prescribed dose during the observation period was slightly higher in males, particularly in those aged ≥75 years. Factors affecting the pharmacokinetics of levodopa include gender, age, and weight/body size. It has been reported that lower body weight tends to result in higher plasma concentrations of levodopa, which may necessitate dose adjustments based on weight, particularly for females who typically weigh less than males (21, 22). Furthermore, even when normalizing body weight, females have greater bioavailability of levodopa and lower drug clearance, which contribute to their increased susceptibility to levodopa-induced dyskinesias (12, 13, 21). The slightly lower maximum dose of levodopa in elderly female patients observed in this study may be explained by these gender differences in levodopa pharmacodynamics and body size. On the other hand, a large multicenter study in North America reported that the levodopa equivalent daily dose of anti-PD drugs, including levodopa, prescribed to early PD patients showed no gender differences when adjusted for disease duration, severity, and weight (23). Consideration of factors such as the type of anti-PD drugs and the severity and duration of PD, in addition to age, gender, and weight, may lead to the implementation of more optimal treatment strategies.

In our study, no large differences were observed in the proportion of levodopa prescriptions between genders, but differences were noted in the types of drugs prescribed following levodopa and those used in combination with levodopa monotherapy. For all patients, non-ergot DA, MAOBI, zonisamide, and catechol-O-methyltransferase inhibitors were commonly prescribed alongside levodopa for both genders. Among these, MAOBI was more frequently prescribed to males across all age groups. Additionally, droxidopa was more commonly prescribed to males aged ≥75 years, while non-ergot DA was more frequently prescribed to females in this age group. For newly treated patients, MAOBI, non-ergot DA, and zonisamide were commonly added to levodopa for both genders, with a notable gender difference in the prescription of MAOBI, particularly in patients aged ≥75 years. One possible reason for the higher prescription rate of MAOBI in males could be the consideration of certain side effects of non-ergot DA, such as somnolence and sudden sleep onset, which may hinder daily activities like work and driving (24). The prescription of droxidopa, which is commonly used for treating neurogenic orthostatic hypotension (25) and freezing of gait (26), was higher in elderly patients compared with younger patients. The particularly high prescription rate among elderly male patients suggests that aging and male gender, which are risk factors for orthostatic hypotension (27), may contribute to this trend. Istradefylline is used as an add-on therapy to levodopa for patients with wearing-off symptoms, and it has recently been reported to have an effect on suppressing the increase in levodopa dosage in Japanese patients (28). It is possible that in males, who tend to have higher levodopa dosages, add-on of istradefylline is aimed at improving motor function without further increasing levodopa dosage. Zonisamide is approved in Japan as a treatment for PD, and it is expected to improve motor symptoms, wearing-off, and tremor when used as an adjunct to levodopa (29). At doses used for PD treatment, zonisamide is less likely to exacerbate dyskinesia and has fewer side effects (30), indicating that it be widely used across genders and ages. In contrast, in newly treated elderly female patients, a broader range of medications following levodopa monotherapy were used without bias toward non-ergot DA, MAOBI, and zonisamide. Multiple studies have highlighted gender differences in various aspects of PD, including motor and non-motor symptoms, and quality of life (31, 32). For instance, depression, sleep disturbances, fatigue, pain, and reduced quality of life are more common in females, who also have a higher incidence of motor complications (11, 31, 33–35). In general, physicians, including movement disorder specialists, develop treatment strategies for PD based on symptoms, severity, efficacy, and side effects of anti-PD drugs. Although these large-scale data may reflect various clinical discretions beyond just specialists, it is suggested that gender differences in the pharmacokinetics and tolerability of levodopa, as demonstrated in previous studies, may be reflected in prescription choices. Our previous study has pointed to the need for treatment strategies in elderly patients that consider the diversity of comorbidities and the decline in metabolic function associated with aging (16). In this study, in addition to age, the impact of gender differences on prescribing patterns was observed. In recent years, an increased prevalence of PD among Japanese females has been reported (36–41). Given the aging population in Japan, the number of elderly female patients with PD is expected to continue to rise. Our findings suggest that in elderly females, who are the major demographic of patients with PD in Japan, treatment choices may reflect gender differences in response to levodopa, body size, and symptomatology, indicating a more tailored approach to treatment. While further studies are needed to investigate gender differences in PD symptoms and treatment, our results could provide important insights for more patient-centered care.

A strength of this study is that it utilizes data from three nationwide healthcare claims databases, providing a comprehensive overview of prescribing patterns across a large population of patients with PD in Japan. Additionally, this analysis includes elderly patients aged 75 years and older, highlighting prescribing trends in a demographic that is often underrepresented in clinical studies, thereby enhancing the relevance of the findings to the aging population.

The limitations of this analysis include the reliance on healthcare claims data, which restricts the ability to confirm actual medication use and does not provide information on the severity or duration of PD, or on clinical outcomes such as treatment efficacy and quality of life. This lack of data may confound the results, particularly in understanding how gender differences might influence treatment choices based on PD severity and comorbidities, which affect treatment strategies. Gender differences in prescribing patterns for individual drugs within each drug class have not been considered. Prospective studies and real-world studies using data from electronic medical records, evaluating symptom severity and clinical outcomes, could complement this study. Additionally, because the data were combined from three nationwide claims databases, the analysis population may not accurately reflect the gender distribution of patients with PD in Japan, limiting the generalizability of the findings. Lastly, the limited observation period of 3–4 years for the databases restricted the ability to assess long-term prescription patterns and characteristics.

## 5 Conclusion

This gender-stratified analysis using healthcare claims data revealed a high proportion of elderly female patients with PD in Japan. These patients received slightly lower maximum levodopa doses and showed less preference for the second-line drug, suggesting a more tailored treatment approach that considers individual patient conditions and gender differences.

## Data Availability

The data analyzed in this study is subject to the following licenses/restrictions: The data that support the findings of this study are available from DeSC Healthcare, Inc. and Japan Medical Data Center, Ltd. but were used under license for the current study; therefore, restrictions apply, and the data are not publicly available. Requests to access these datasets should be directed to DeSC Healthcare, Inc. (https://desc-hc.co.jp/en) and Japan Medical Data Center Ltd. (https://www.jmdc.co.jp/en/).

## References

[1] Hirsch L Jette N Frolkis A Steeves T Pringsheim T. The incidence of Parkinson's disease: a systematic review and meta-analysis. Neuroepidemiology. (2016) 46:292–300. doi: 10.1159/000445751, PMID: 27105081

[2] Dorsey ER Sherer T Okun MS Bloem BR. The emerging evidence of the Parkinson pandemic. J Parkinsons Dis. (2018) 8:S3–8. doi: 10.3233/JPD-181474, PMID: 30584159 PMC6311367

[3] Zafar S Yaddanapudi SS. Parkinson disease, StatPearls. Treasure Island, FL: StatPearls Publishing (2025). 29261972

[4] Poewe W Seppi K Tanner CM Halliday GM Brundin P Volkmann J. Parkinson disease. Nat Rev Dis Primers. (2017) 3:17013. doi: 10.1038/nrdp.2017.13, PMID: 28332488

[5] Japanese Society of Neurology. Parkinson’s disease treatment guidelines 2018. Available online at: https://www.neurology-jp.org/guidelinem/parkinson_2018.html (Accessed October 4, 2024).

[6] Miller IN Cronin-Golomb A. Gender differences in Parkinson's disease: clinical characteristics and cognition. Mov Disord. (2010) 25:2695–703. doi: 10.1002/mds.23388, PMID: 20925068 PMC3003756

[7] Haaxma CA Bloem BR Borm GF Oyen WJ Leenders KL Eshuis S. Gender differences in Parkinson's disease. J Neurol Neurosurg Psychiatry. (2007) 78:819–24. doi: 10.1136/jnnp.2006.103788, PMID: 17098842 PMC2117736

[8] Perrin AJ Nosova E Co K Book A Iu O Silva V. Gender differences in Parkinson's disease depression. Parkinsonism Relat Disord. (2017) 36:93–7. doi: 10.1016/j.parkreldis.2016.12.026, PMID: 28089265

[9] Silverdale MA Kobylecki C Kass-Iliyya L Martinez-Martin P Lawton M Cotterill S. A detailed clinical study of pain in 1957 participants with early/moderate Parkinson's disease. Parkinsonism Relat Disord. (2018) 56:27–32. doi: 10.1016/j.parkreldis.2018.06.001, PMID: 29903584 PMC6302227

[10] Broen MPG Leentjens AFG Hinkle JT Moonen AJH Kuijf ML Fischer NM. Clinical markers of anxiety subtypes in Parkinson disease. J Geriatr Psychiatry Neurol. (2018) 31:55–62. doi: 10.1177/0891988718757369, PMID: 29528763 PMC5903060

[11] Cerri S Mus L Blandini F. Parkinson's disease in women and men: what's the difference? J Parkinsons Dis. (2019) 9:501–15. doi: 10.3233/JPD-191683, PMID: 31282427 PMC6700650

[12] Contin M Lopane G Belotti LMB Galletti M Cortelli P Calandra-Buonaura G. Sex is the main determinant of levodopa clinical pharmacokinetics: evidence from a large series of levodopa therapeutic monitoring. J Parkinsons Dis. (2022) 12:2519–30. doi: 10.3233/JPD-223374, PMID: 36373294 PMC9837688

[13] Conti V Izzo V Russillo MC Picillo M Amboni M Scaglione CLM. Gender differences in levodopa pharmacokinetics in levodopa-naïve patients with Parkinson's disease. Front Med (Lausanne). (2022) 9:909936. doi: 10.3389/fmed.2022.909936, PMID: 35712091 PMC9193593

[14] Orayj K Lane E. Patterns and determinants of prescribing for Parkinson's disease: a systematic literature review. Parkinsons Dis. (2019) 2019:9237181. doi: 10.1155/2019/9237181, PMID: 31781365 PMC6875178

[15] Suzuki M Arai M Hayashi A Ogino M. Adherence to treatment guideline recommendations for Parkinson's disease in Japan: a longitudinal analysis of a nationwide medical claims database between 2008 and 2016. PLoS One. (2020) 15:e0230213. doi: 10.1371/journal.pone.0230213, PMID: 32330133 PMC7182259

[16] Seki M Kawata Y Hayashi A Arai M Fujimoto S. Prescribing patterns and determinants for elderly patients with Parkinson's disease in Japan: a retrospective observational study using insurance claims databases. Front Neurol. (2023) 14:1162016. doi: 10.3389/fneur.2023.1162016, PMID: 37426443 PMC10327598

[17] Ministry of Health, Labour and Welfare. Policy information: an outline of the Japanese medical system. Available online at: https://www.mhlw.go.jp/bunya/iryouhoken/iryouhoken01/dl/01_eng.pdf (Accessed May 14, 2025).

[18] Actuarial Research Division, Health Insurance Bureau, Ministry of Health, Labour and Welfare. Basic data on medical insurance—status of medical expense, etc. in the fiscal year of 2021 [in Japanese] Available online at: https://www.mhlw.go.jp/content/kiso_r03.pdf (Accessed May 14, 2025).

[19] Ministry of Health, Labour and Welfare. Overview of the 2020 patient survey [in Japanese]. Available online at: https://www.mhlw.go.jp/toukei/saikin/hw/kanja/20/index.html (Accessed November 4, 2024).

[20] Takeda A Baba T Watanabe J Nakayama M Hozawa H Ishido M. Levodopa prescription patterns in patients with advanced Parkinson's disease: a Japanese database analysis. Parkinsons Dis. (2023) 2023:9404207. doi: 10.1155/2023/9404207, PMID: 37799489 PMC10550461

[21] Nishikawa N Iwaki H Shiraishi T Mukai Y Takahashi Y Murata M. Female, aging, difference formulations of DCI, or lower body weight increases AUC_4hr_ of levodopa in patients with Parkinson's disease. Parkinsonism Relat Disord. (2020) 76:16–20. doi: 10.1016/j.parkreldis.2020.05.020, PMID: 32554330

[22] Zappia M Crescibene L Arabia G Nicoletti G Bagalà A Bastone L. Body weight influences pharmacokinetics of levodopa in Parkinson's disease. Clin Neuropharmacol. (2002) 25:79–82. doi: 10.1097/00002826-200203000-00004, PMID: 11981233

[23] Umeh CC Pérez A Augustine EF Dhall R Dewey RB Jr Mari Z. No sex differences in use of dopaminergic medication in early Parkinson disease in the US and Canada - baseline findings of a multicenter trial. PLoS One. (2014) 9:e112287. doi: 10.1371/journal.pone.0112287, PMID: 25486269 PMC4259292

[24] Di Laudo F Baldelli L Mainieri G Loddo G Montini A Pazzaglia C. Daytime sleepiness in Parkinson's disease: a multifaceted symptom. Front. Sleep. (2023) 2:1302021. doi: 10.3389/frsle.2023.1302021, PMID: 40530011

[25] Hauser RA Isaacson S Lisk JP Hewitt LA Rowse G. Droxidopa for the short-term treatment of symptomatic neurogenic orthostatic hypotension in Parkinson's disease (nOH306B). Mov Disord. (2015) 30:646–54. doi: 10.1002/mds.26086, PMID: 25487613

[26] Narabayashi H Nakanishi T Yoshida M Yanagisawa N Mikuni M Kanazawa I. Therapeutic effect of L-DOPS in Parkinson’s disease: a double-blind comparative study using placebo in patients with levodopa baseline therapy [in Japanese]. Rinsho Hyoka. (1987) 15:423–57.

[27] Fereshtehnejad SM Lökk J. Orthostatic hypotension in patients with Parkinson's disease and atypical parkinsonism. Parkinsons Dis. (2014) 2014:475854. doi: 10.1155/2014/475854, PMID: 24634790 PMC3929346

[28] Hatano T Sengoku R Nagayama H Yanagisawa N Yoritaka A Suzuki K. Impact of istradefylline on levodopa dose escalation in Parkinson's disease: ISTRA ADJUST PD study, a multicenter, open-label, randomized, parallel-group controlled study. Neurol Ther. (2024) 13:323–38. doi: 10.1007/s40120-023-00574-6, PMID: 38227133 PMC10951171

[29] Zesiewicz TA Elble RJ Louis ED Gronseth GS Ondo WG Dewey RB Jr. Evidence-based guideline update: treatment of essential tremor: report of the Quality Standards Subcommittee of the American Academy of Neurology. Neurology. (2011) 77:1752–5. doi: 10.1212/WNL.0b013e318236f0fd, PMID: 22013182 PMC3208950

[30] Murata M Hasegawa K Kanazawa I Japan Zonisamide on PD Study Group. Zonisamide improves motor function in Parkinson disease: a randomized, double-blind study. Neurology. (2007) 68:45–50. doi: 10.1212/01.wnl.0000250236.75053.16, PMID: 17200492

[31] Russillo MC Andreozzi V Erro R Picillo M Amboni M Cuoco S. Sex differences in Parkinson's disease: from bench to bedside. Brain Sci. (2022) 12:917. doi: 10.3390/brainsci12070917, PMID: 35884724 PMC9313069

[32] Subramanian I Mathur S Oosterbaan A Flanagan R Keener AM Moro E. Unmet needs of women living with Parkinson's disease: gaps and controversies. Mov Disord. (2022) 37:444–55. doi: 10.1002/mds.28921, PMID: 35060180

[33] Martinez-Martin P Falup Pecurariu C Odin P van Hilten JJ Antonini A Rojo-Abuin JM. Gender-related differences in the burden of non-motor symptoms in Parkinson's disease. J Neurol. (2012) 259:1639–47. doi: 10.1007/s00415-011-6392-3, PMID: 22237822

[34] Santos-García D Laguna A Hernández-Vara J de Deus Fonticoba T Cores Bartolomé C Feal Painceiras MJ. Sex differences in motor and non-motor symptoms among Spanish patients with Parkinson's disease. J Clin Med. (2023) 12:1329. doi: 10.3390/jcm12041329 36835866 PMC9960095

[35] Wan Z Wang X Ma H Wang Z Feng T. Risk factors for motor complications in female patients with Parkinson's disease. Neurol Sci. (2022) 43:4735–43. doi: 10.1007/s10072-022-05959-3, PMID: 35384562

[36] Kimura H Kurimura M Wada M Kawanami T Kurita K Suzuki Y. Female preponderance of Parkinson's disease in Japan. Neuroepidemiology. (2002) 21:292–6. doi: 10.1159/000065527, PMID: 12411732

[37] Kusumi M Nakashima K Harada H Nakayama H Takahashi K. Epidemiology of Parkinson's disease in Yonago City, Japan: comparison with a study carried out 12 years ago. Neuroepidemiology. (1996) 15:201–7. doi: 10.1159/000109908, PMID: 8817502

[38] Morioka S Sakata K Yoshida S Nakai E Shiba M Yoshimura N. Incidence of Parkinson disease in Wakayama, Japan. J Epidemiol. (2002) 12:403–7. doi: 10.2188/jea.12.403, PMID: 12462274 PMC10681818

[39] Moriwaka F Tashiro K Itoh K Honma S Okumura H Kikuchi S. Prevalence of Parkinson's disease in Hokkaido, the northernmost island of Japan. Intern Med. (1996) 35:276–9. doi: 10.2169/internalmedicine.35.276, PMID: 8739781

[40] Okada K Kobayashi S Tsunematsu T. Prevalence of Parkinson's disease in Izumo City, Japan. Gerontology. (1990) 36:340–4. doi: 10.1159/000213219, PMID: 2076832

[41] Osaki Y Morita Y Kuwahara T Miyano I Doi Y. Prevalence of Parkinson's disease and atypical parkinsonian syndromes in a rural Japanese district. Acta Neurol Scand. (2011) 124:182–7. doi: 10.1111/j.1600-0404.2010.01442.x, PMID: 20880268

