# Supplementary material for: Gender differences in prescribing patterns for patients with Parkinson’s disease in Japan: a retrospective observational study using insurance claims databases
Source: Front Neurol. 2025 Jul 4;16:1571718. doi: 10.3389/fneur.2025.1571718 (PMC12273449; doi:10.3389/fneur.2025.1571718)
Supplement: Supplementary file 1 [file Supplementary_file_1.docx]

SUPPLEMENTARY MATERIAL

**
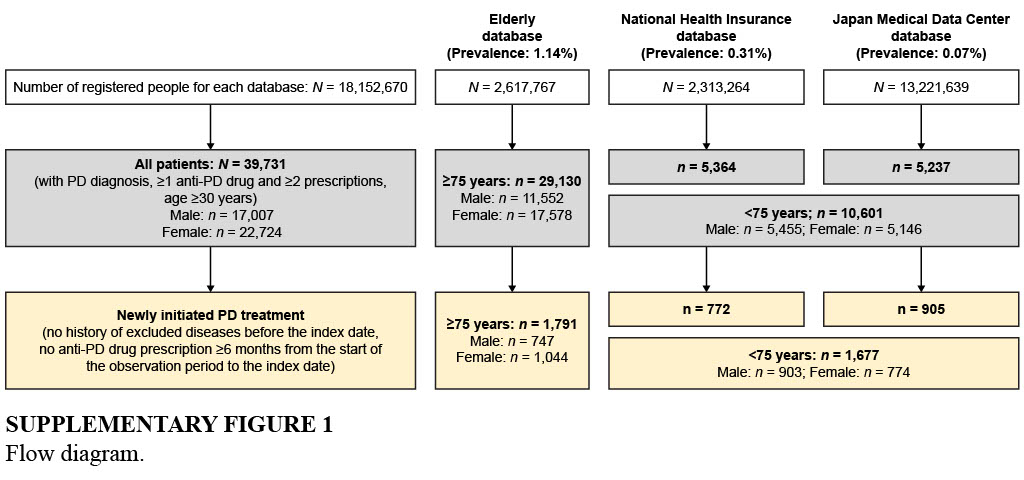
**

**SUPPLEMENTARY Table 1** **Classification of drugs prescribed for PD**

| **Drug** | **Receipt code from computerized processing system** | **ATC code** |
| --- | --- | --- |
| Levodopa  Levodopa  Levodopa carbidopa hydrate  Levodopa benserazide hydrochloride  Levodopa carbidopa hydrate entacapone | 620006042, 620006043, 620008660, 620008661, 620008662, 620008889, 620008890 | N04BA01 |
|  | 620123601, 620123701, 620123801, 620123901, 620124001, 620124101, 620124201, 620124301, 620124401, 620124501, 620124601, 621865201, 622050801, 622051301, 622067101, 622080201, 622509401, 622711600, 622711800 | N04BA02 |
|  | 620008030, 620008659, 622325500, 622711700 | N04BA02 |
|  | 622375701, 622375801 | N04BA03 |
| Dopamine agonist – ergot  Bromocriptine mesilate  Pergolide mesilate  Cabergoline | 610422106, 620005063, 620005096, 620006069, 620006743, 620008579, 620008688, 620009237, 620120902, 620121001, 620121101, 620121102, 620121401, 620121701, 620121802, 620121902, 620121904, 620122001 | N04BC01 |
|  | 620121801 | NA |
|  | 610406392, 610406393, 620002124, 620002125, 620002823, 620002857, 620002858, 620004067, 620004068, 621642002, 621642103, 621690101 | N04BC02 |
|  | 620006684, 620006685, 622711100, 622711200 | NA |
|  | 610432023, 610432024, 620005369, 620005370, 620005371, 620005372, 620005375, 620005376, 620005377, 620005378, 620005379, 620008002, 622711400, 622711500 | N04BC06 |
|  | 620005373, 620006667, 620006668, 620008001 | NA |
| Dopamine agonist – non-ergot  Talipexole hydrochloride  Ropinirole  Pramipexole hydrochloride hydrate  Apomorphine hydrochloride hydrate  Rotigotine | 610409337 | N04BC |
|  | 620004415, 620004416, 620004417, 622183601, 622183701, 622492301, 622492401, 622492501, 622500001, 622500101, 622500201, 622582201, 622582301, 622583401, 622583501, 622656201, 622656301, 622687201, 622687301, 622687401, 622687501, 622687601 | N04BC04 |
|  | 620000435, 620000436, 622069601, 622069701, 622226701, 622226801, 622228701, 622228801, 622229801  622229901, 622231601, 622231701, 622233901, 622234001, 622235901, 622236001, 622238001, 622238101, 622239601, 622239701, 622245201, 622245301, 622247301, 622247401, 622249201, 622249301, 622253101, 622253201, 622253501, 622253601, 622254501, 622254601, 622256401, 622256501, 622257401, 622257501, 622272601, 622272701, 622511201, 622511301, 622513801, 622513901, 622520201, 622520301, 622524801, 622524901, 622528201, 622528301, 622530001, 622530101, 622691900 | N04BC05 |
|  | 622263001, 622263101, 622263201, 622263301, 622692000 | NA |
|  | 622149301 | N04BC07 |
|  | 622163501, 622163601, 622163701, 622163801, 622478301 | N04BC09 |
| Monoamine oxidase type B inhibitor  Selegiline hydrochloride  Rasagiline mesilate  Safinamide mesilate | 610421338, 620003975, 620005364, 620005511, 620008494, 622711300 | N04BD01 |
|  | 622624401, 622624501 | N04BD02 |
|  | 622696101 | N04BD03 |
| Catechol-*O*-methyltransferase inhibitor  Entacapone  Opicapone | 620004853, 622478401, 622490101, 622498501, 622503001 | N04BX02 |
|  | 622845500, 622878901 | NA |
|  | 622821701 | N04BX04 |
| Anticholinergic agent  Trihexyphenidyl hydrochloride  Biperiden hydrochloride  Biperiden lactate  Profenamine hydrochloride  Profenamine hibenzate | 610454002, 611240304, 611240415, 611240423, 611240425, 620003078, 620004382, 620004384, 620004548, 620006589, 620008311, 620008663, 620119903, 620119906, 620119907, 620119919, 620119923, 620119927, 620120303 | N04AA01 |
|  | 620004556, 620005847, 622319200 | NA |
|  | 610444006, 610444007, 620000066, 620000067, 620000243, 620004893, 620004894, 620005147, 620117802, 620118102, 620118203, 620118402, 621218501, 621392101 | N04AA02 |
|  | 620006075 | NA |
|  | 611240172, 611240173 | N04AA05 |
|  | 610463153 |  |
| Zonisamide | 611130087, 620001972, 620009098, 621390801, 621390901, 622400401, 622589801, 622673201, 622709200, 622709300 | N03AX15 |
| Istradefylline | 622245801 | N04CX01 |
| Amantadine | 610461044, 610461047, 611240079, 611240080, 620002335, 620004505, 620008286, 620116802, 620116809, 620117101, 620117103, 620117110, 620117112, 620117114, 620117401, 620117402, 620117404, 620117412, 620117413, 620117414, 622710800 | N04BB01 |
|  | 610461046, 611240122, 611240123, 620003077, 620003267, 620003268, 620005903, 620006587, 620008280, 620116701, 620117107, 620117410, 620116702, 622309500, 620116805 | NA |
| Droxidopa | 620004007, 620004008, 620004009, 620004010, 620005044, 620005045, 620005046, 620006676, 620006677, 620006678, 622061101, 622061201 | C01CA27 |
|  | 622710900, 622711000 |  |

ATC, Anatomical Therapeutic Chemical; NA, not applicable; PD, Parkinson’s disease.

This table was previously reported in Seki M, et al. Front Neurol (2023) 14:1162016. doi: 10.3389/fneur.2023.1162016, licensed under CC BY 4.0 (<https://creativecommons.org/licenses/by/4.0/>).
